# Comparison of non-insulin antidiabetic agents as an add-on drug to insulin therapy in type 2 diabetes: a network meta-analysis

**DOI:** 10.1038/s41598-018-22443-1

**Published:** 2018-03-06

**Authors:** Jeong-Hwa Yoon, Se Hee Min, Chang Ho Ahn, Young Min Cho, Seokyung Hahn

**Affiliations:** 1 Interdisciplinary Program in Medical Informatics, Seoul National University College of Medicine, Seoul, South Korea; 2 Division of Endocrinology and Metabolism, Department of Internal Medicine, Seoul National University College of Medicine, Seoul, South Korea; 3 Department of Medicine, Seoul National University College of Medicine, Seoul, South Korea

## Abstract

We aimed to evaluate the comparative efficacy and safety of dipeptidyl peptidase-4 inhibitors (DPP4i), glucagon-like peptide-1 receptor agonists (GLP-1RA), sodium-glucose co-transporter 2 inhibitors (SGLT2i), or thiazolidinedione (TZD) as an adjunctive treatment in patients with poorly controlled type 2 diabetes mellitus (T2DM) on insulin therapy. We searched Medline, Embase, the Cochrane Library, and ClinicalTrials.gov through April 2016. Bayesian network meta-analyses were performed with covariate adjustment. The primary outcome was the change in glycated hemoglobin A1c (HbA1c) from baseline. Fifty randomized controlled trials covering 15,494 patients were included. GLP-1RA showed the greatest HbA1c-lowering effect compared to the control (−0.84%; 95% credible interval, −1.00% to −0.69%), followed by TZD (−0.73%; −0.93 to −0.52%), SGLT2i (−0.66%; −0.84% to −0.48%), and DPP4i (−0.54%; −0.68% to −0.39%). SGLT2i showed the greatest fasting plasma glucose reduction. GLP-1RA and SGLT2i showed greater body weight reduction, whereas TZD increased body weight. TZD was ranked the highest in terms of insulin dose reduction. The risk of hypoglycemia was increased with TZD or GLP-1RA. The study provides the best available evidence on the comparative efficacy and safety of non-insulin anti-diabetic agents on top of pre-existing insulin therapy for inadequately controlled T2DM patients.

## Introduction

Impaired insulin secretion in the presence of insulin resistance is the key feature of type 2 diabetes mellitus (T2DM). The progressive nature of insulin secretory failure makes T2DM patients require insulin therapy to achieve their glycemic goals^1^, but intensifying insulin therapy increases the risk of hypoglycemia and weight gain^2^. Therefore, as an alternative to more intensive insulin therapy, a non-insulin anti-diabetic drug may be used as an add-on drug to insulin therapy. In this regard, recent guidelines for T2DM management have recommended the use of dipeptidyl peptidase-4 inhibitors (DPP4i), glucagon-like peptide-1 receptor agonists (GLP-1RA), sodium-glucose co-transporter 2 inhibitors (SGLT2i), or thiazolidinedione (TZD) on top of pre-existing insulin therapy, particularly basal insulin^3,4^. Each agent has both advantages and disadvantages according to the characteristics of the drug, which need to be considered when determining the most appropriate treatment for an individual patient. To the best of our knowledge, there has been no comparative efficacy or effectiveness study of adjunctive treatments to insulin therapy. Hence, we performed a systematic review with a network meta-analysis to evaluate the comparative efficacy and safety of DPP4i, GLP-1RA, SGLT2i, and TZD as an adjunctive treatment in patients with poorly controlled T2DM on insulin therapy.

## Methods

We conducted a systematic review and network meta-analysis following a pre-developed protocol (Supplementary Appendix 1).

### Search strategy and study selection

The following electronic databases were searched from inception to April 2016: Medline, Embase, Cochrane Central Register of Controlled Trials (CENTRAL), and ClinicalTrials.gov. The detailed search strategy used in Medline is available in Supplementary Appendix 2.

We included randomized controlled trials (RCTs) that investigated the effect of DPP4i, GLP-1RA, SGLT2i, or TZD as an add-on drug to pre-existing insulin therapy in patients with T2DM. Both placebo-controlled and open-label trials were included, and concurrent use of other anti-diabetic agents was allowed. Studies reporting the change of HbA1c from baseline were included when some information on the mean and its variability was available. An intervention of at least 12 weeks was required and studies in English were considered for inclusion. Duplicate publications or studies on post-hoc analysis were excluded. We also excluded studies with patients treated using an insulin pump. Two reviewers (J.-H.Y. and S.H.M.) independently screened all studies by title and abstract, and then by full text, to assess the eligibility of the studies. Any discrepancies between the authors were resolved through mutual discussions with the other authors (C.H.A., Y.M.C., and S.H.).

### Data extraction

We extracted the following information from each study: study information, participant characteristics at baseline, information on interventions and values of outcome variables (as a primary outcome, the change in HbA1c from baseline; as secondary efficacy outcomes, the change in fasting plasma glucose [FPG] levels, body weight, insulin dose and the proportion of patients achieving HbA1c goals; and as a safety outcome, the risk of hypoglycemia). We attempted to classify various insulin regimens into 2 categories: a stable insulin dose group, in which the insulin doses were kept relatively constant throughout the study duration unless the dose needed to be altered for safety reasons, and an insulin dose titration group, in which the insulin doses were titrated according to study-specific predefined titration algorithms. We obtained information from the ClinicalTrials.gov website when the studies had never been published as an article.

### Assessment of the study quality and risk of bias

Two independent reviewers (J.-H.Y. and S.H.M.) assessed the study quality and risk of bias according to the Cochrane Collaboration tool^5^, and any controversies were resolved by mutual discussion. We considered the 6 aspects of risk of bias, including the adequacy of random sequence generation, allocation concealment, blinding of participants and personnel, completeness of outcome data, selective reporting, and other sources of bias. For the completeness of outcome data, analyses based on the intention-to-treat principle or the full analysis set were considered to be low-risk. Selective reporting was assessed as high-risk when any results were regarded as missing while some outcome variables or conditions of analysis were mentioned in the articles or when the publication was not considered as in full. Other sources of bias were assessed according to the comparability of the baseline characteristics of the participants in the trial groups.

### Statistical analysis

For the continuous variables, pooled weighted mean differences between treatment groups were calculated. For the dichotomous variables, we calculated the pooled relative risks (RR). Prior to conducting the network meta-analysis, homogeneity in the common comparator (insulin therapy with placebo add-on or open-label; hereafter, ‘control group’ collectively) was assessed as an indicator of study comparability^6,7^. This was done primarily by a qualitative review of the regimen used in each study, and by a quantitative comparison of the results from the control group in all included studies using the ${\rm{\chi }}$^2^ test and I^2^ statistics, with a forest plot presenting the pooled weighted mean and the 95% confidence intervals (CIs). We considered that participants’ age, sex, body mass index (BMI), baseline HbA1c, duration of diabetes, and baseline insulin dose could affect within-group or between-group heterogeneity, and therefore could be potential confounders. Although we reviewed each insulin titration algorithm used in all the included studies, it was not possible to include it as a covariate for adjustment due to the diversity of algorithms. However, since the treatment results from the control group inherently reflect the effects of various insulin regimens, we used them as a modifier representing unmeasured prognostic factors related to various insulin regimens across the included trials^8^. We explored the potential relationship between each candidate covariate and the outcome by a scatter plot with a conventional random-effects meta-regression. The network meta-analysis was conducted using a Bayesian approach, and the posterior distribution for each parameter of interest was summarized with a median and the 95% credible interval (CrI)^9,10^. We selected covariates that contributed to the network meta-analysis, including each as a covariate at a time, based on the 95% CrI of the coefficient. Covariates with a strong clinical rationale were also selected. The final network meta-analysis was adjusted for the selected covariates with a random-effects model. We performed sensitivity analyses after mean imputation of the values of covariates missing in some studies, and additionally by including only placebo-controlled trials. We estimated the surface under the cumulative ranking curve^11^ for each treatment for each outcome, which provides the probability for each treatment to be ranked as the most effective treatment or to have the highest chance for a respective adverse event. We used STATA version 12 (Stata Corp, College Station, TX, USA), R version 3.2.1 (R Foundation for Statistical Computing, Vienna, Austria), and WinBUGS version 14 (Imperial College and Medical Research Council, London, United Kingdom).

### Data availability

The datasets generated during and/or analyzed during the current study are available from the corresponding author on reasonable request.

## Results

### Search results and study characteristics

**Figure 1:**
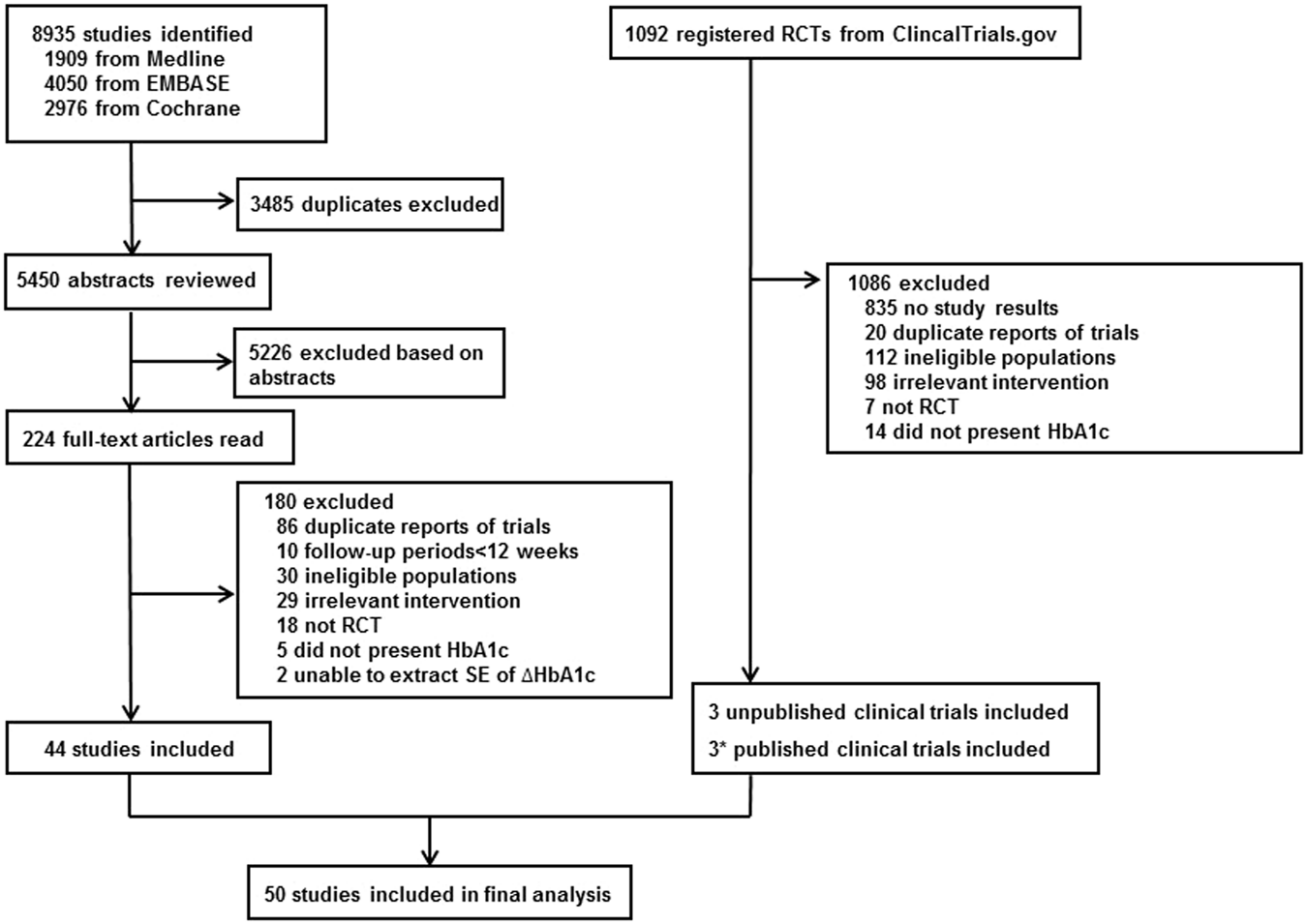
Study selection process. RCT, randomized controlled trial; SE, standard error. The asterisk indicates that 3 trials of 6 previously unpublished studies were published after literature search.

**Figure 2:**
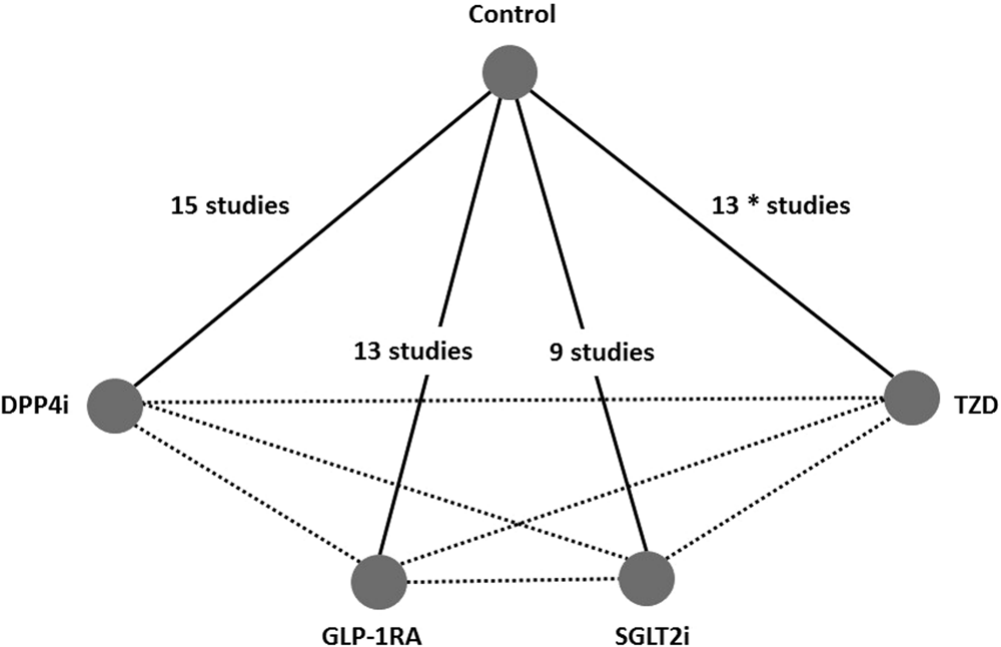
Structure of the network formed by interventions and both direct and indirect comparisons for primary outcomes. Solid lines and dashed lines indicated direct and indirect comparisons, respectively. The numbers next to each solid line joining two groups correspond to the number of studies that compared those groups. The asterisk indicates that 1 of the 13 trials was a 3-arm trial that compared 2 different kinds of TZD with the control. Control, insulin therapy with placebo add-on or open-label; GLP-1RA, glucagon-like peptide-1 receptor agonists plus insulin; DPP4i, dipeptidyl peptidase-4 inhibitor plus insulin; SGLT2i, sodium-glucose co-transporter 2 inhibitor plus insulin; TZD, thiazolidinedione plus insulin.

The flowchart of the literature selection is shown in Fig. 1. We retrieved 8935 potentially relevant studies, of which 44 articles were finally included in the analysis^12–55^. We additionally selected 6 trials^56–61^ among the 1092 clinical trials identified from ClinicalTrials.gov, of which 3 trials^57–59^ were unpublished. The network frame based on the 50 studies is depicted in Fig. 2. There were 15 DPP4i^15,22,23,26,29–31,34,38,45,46,50,55,57,61^, 13 GLP-1RA^12,16,18,20,21,32,33,41,42,47,48,56,59^, 9 SGLT2i^13,19,37,43,44,51,52,58,60^ and 13 TZD^14,17,24,25,27,28,35,36,39,40,49,53,54^ studies included in the network. No study with head-to-head comparisons between the non-insulin agents was found. The characteristics of the included trials are described in Table 1. Fifty studies with a mean study duration of 23 weeks included 15,494 randomized participants. The patients’ overall mean age was 58.7 years and the age distribution was similar in all treatment groups. The mean baseline HbA1c ranged from 7.3% to 9.8% among studies. The mean duration of diabetes was approximately 13 years, and was similar in most of the studies. The mean baseline insulin dose was 54.6 units per day over all studies. Twenty-six studies^12,13,15,19,22,25,26,29–31,37–41,43–45,47,48,50–52,55,60,61^ were considered as having a stable insulin dose, whereas 19 studies^14,16–18,20,21,24,27,28,32–35,42,46,49,53,54,56^ involved insulin dose titration. The rest^23,36,57–59^ of the studies could not be classified in either group because they did not describe the method of insulin dosage. Two studies^33,56^ with fixed ratio combination products of GLP-1RA and insulin were included.

**Table 1:** Summary of the studies included in the network meta-analysis.

Study source	*Study duration of primary phase, weeks*	Insulin regimen	Study arms	Randomized participants, N	Age, years	Proportion of males, %	Baseline BMI, kg/m^2^	Baseline HbA1c, %	Duration of diabetes, years	Baseline insulin dose, IU/day
*Insulin plus DPP4 inhibitor*										
Barnett *et al*.^15^	24	Stable	saxagliptin 5 mg + insulin ± metformin	304	57.2	40.0	32.6	8.7	11.8	53.6
			placebo + insulin ± metformin	151	57.3	45.0	31.8	8.6	12.2	55.3
Fonseca *et al*.^22^	24	Stable	vildagliptin 50 mg bid + insulin	144	59.6	47.9	33.3	8.4	14.4	81.2
			placebo + insulin	152	58.9	54.6	32.9	8.4	14.9	81.9
Franc *et al*.^23^	12	NR	vildagliptin 50 mg bid + insulin + metformin	31	59.4	NR	28.6	7.7	6.1	39.3
			placebo + insulin + metformin	31	59.4	NR	28.6	7.7	6.1	39.3
Hirose *et al*.^26^	12	Stable	vildagliptin 50 mg bid + insulin ± metformin	78	58.5	70.5	25.3	8.1	12.8	20.7
			placebo + insulin ± metformin	78	60.1	71.8	26.0	8.1	12.7	21.1
Kadowaki *et al*.^29^	16	Stable	sitagliptin 50 mg qd + insulin	129	62.3	58.9	25.2	8.9	14.1	24.4
			placebo + insulin	137	60.2	58.4	25.2	8.9	14.0	24.3
Kaku *et al*.^30^	12	Stable	alogliptin 25 mg qd + insulin	90	62.9	55.6	23.9	8.4	15.3	22.6
			placebo + insulin	89	62.4	52.8	24.7	8.4	14.5	23.7
Kothny *et al*.^31^	24	Stable	vildagliptin 50 mg bid + insulin ± metformin	228	59.3	47.8	28.9	8.8	12.9	39.9
			placebo + insulin ± metformin	221	59.1	52.0	29.0	8.8	13.2	41.9
Mathieu *et al*.^34^	24	Titration	sitagliptin 100 mg per day + glargine ± metformin	329	59.3	45.9	31.9	8.7	13.2	37.3
			placebo + glargine ± metformin	329	58.3	49.8	32.3	8.8	13.7	36.6
Ning *et al*.^38^	24	Stable	vildagliptin 50 mg bid + insulin ± metformin	146	57.8	41.8	26.2	8.6	11.2	33.3
			placebo + insulin ± metformin	147	58.4	44.9	26.0	8.7	11.4	31.7
Rosenstock *et al*.^45^	26	Stable	alogliptin 25 mg qd + insulin ± metformin	129	55.9	34.0	32.3	9.3	13.4	55.0
			placebo + insulin ± metformin	130	55.0	48.0	32.4	9.3	12.2	57.0
Sato *et al*.^46^	24	Titration	sitagliptin 50 mg or 100 mg qd + insulin ± OADs	25	66.0	64.0	24.5	7.9	19.0	31.8
			insulin ± OADs	24	66.0	75.0	26.8	7.8	20.0	32.5
Shankar *et al*.^61^	24	Stable	sitagliptin 100 mg qd + insulin ± metformin	234	58.6	55.6	25.9	8.7	11.0	34.5
			placebo + insulin ± metformin	233	56.7	49.8	26.1	8.8	11.3	34.5
Vilsboll *et al*.^50^	24	Stable	sitagliptin 100 mg qd + insulin ± metformin	322	58.3	49.0	31.0	8.7	13.0	44.2
			placebo + insulin ± metformin	319	57.2	53.0	31.0	8.6	12.0	44.5
Yki-Jarvinen *et al*.^55^	24	Stable	linagliptin 5 mg qd + insulin ± metformin ± pioglitazone	631	59.7	52.1	30.8	8.3	NR	41.5
			placebo + insulin ± metformin ± pioglitazone	630	60.4	52.2	31.2	8.3	NR	40.1
NCT02081599^57^	16	NR	teneligliptin 20 mg qd + insulin	77	NR	76.6	NR	NR	NR	NR
			placebo + insulin	71	NR	74.6	NR	NR	NR	NR
Insulin plus GLP-1 receptor agonist										
Ahmann *et al*.^12^	26	Stable	liraglutide 0.6–1.8 mg qd + insulin ± metformin	225	59.3	53.3	32.3	8.2	12.1	40.5
			placebo + insulin ± metformin	225	57.5	60.4	32.2	8.3	12.1	40.5
Aroda *et al*.^56^	30	Titration	fixed combination (lixisenatide + glargine) ± metformin	367	59.6	45.0	31.3	8.1	12.0	35.0
			glargine ± metformin	369	60.3	48.5	31.0	8.1	12.1	35.2
Buse *et al*.^16^	30	Titration	exenatide 10 μg bid + insulin ± metformin ± pioglitazone	137	59.0	51.0	33.8	8.3	12.0	49.5
			placebo + insulin ± metformin ± pioglitazone	122	59.0	64.0	33.1	8.5	12.0	47.4
Buse *et al*.^18^	26	Titration	liraglutide 0.6–1.8 mg qd + degludec + metformin ± SU/glinides	199	57.0	56.0	33.6	8.7	10.0	29.0
			placebo + degludec + metformin ± SU/glinides	199	58.0	53.0	33.8	8.8	11.0	29.0
De Wit *et al*.^20^	26	Titration	liraglutide 0.6–1.8 mg qd + insulin ± metformin ± SU	26	57.0	61.5	34.0	7.2	8.3	54.0
			insulin ± metformin ± SU	24	59.0	62.5	32.0	7.5	7.6	50.0
Distiller *et al*.^21^	24	Titration	exenatide 10 μg bid + insulin + metformin	14	49.1	50.0	41.9	8.7	12.3	253.0
			insulin + metformin	14	54.3	50.0	40.2	9.2	12.4	237.0
Lind *et al*.^32^	24	Titration	liraglutide 0.6–1.8 mg qd + insulin ± metformin	64	63.7	62.5	33.7	9.0	17.3	105.3
			placebo + insulin ± metformin	60	63.5	66.7	33.5	8.9	17.0	105.7
Lingvay *et al*.^33^	26	Titration	fixed combination (liraglutide 0.6–1.8 mg qd + degludec) ± metformin	278	58.4	51.4	31.7	8.4	11.64	31.0
			glargine ± metformin	279	59.1	49.1	31.7	8.2	11.33	32.0
Riddle *et al*.^41^ (GetGoal-L)	24	Stable	lixisenatide 10–20 μg qd + insulin ± metformin	328	57.0	45.0	31.9	8.4	12.5	54.0
			placebo + insulin ± metformin	167	57.0	49.0	32.6	8.4	12.4	58.0
Riddle *et al*.^42^ (GetGoal-Duo 1)	24	Titration	lixisenatide 10–20 μg qd + glargine + metformin ± TZD	223	56.0	49.0	32.0	7.6	9.6	43.4
			placebo + glargine + metformin ± TZD	223	56.0	51.0	31.7	7.6	8.7	44.2
Seino *et al*.^48^	24	Stable	lixisenatide 20 μg qd + insulin ± SU	154	58.7	44.8	25.4	8.5	13.7	24.9
			placebo + insulin ± SU	157	58.0	51.0	25.2	8.5	14.1	24.1
Seino *et al*.^47^	16	Stable	liraglutide 0.9 mg qd + insulin	127	61.3	54.3	26.2	8.8	14.32	30.0
			placebo + insulin	130	59.8	57.7	25.2	8.8	14.69	29.0
NCT02152371^59^	28	NR	dulaglutide 1.5 mg qw + glargine ± metformin	150	60.2	56.7	NR	NR	NR	40.7
			placebo + glargine ± metformin	150	60.6	58.7	NR	NR	NR	36.6
Insulin plus SGLT2 inhibitor										
Araki *et al*.^13^	16	Stable	dapagliflozin 5 mg qd + insulin ± DPP4i	122	58.3	73.0	26.89	8.3	15.32	37.9
			placebo + insulin ± DPP4i	60	57.6	66.7	26.12	8.5	14.24	40.6
Cefalu *et al*.^19^	24	Stable	dapagliflozin 10 mg qd + insulin ± OADs	234	NR	NR	NR	8.3	NR	56.8
			placebo + insulin ± OADs	242	NR	NR	NR	8.2	NR	49.2
Inagaki *et al*.^60^	16	Stable	canagliflozin 100 mg qd + insulin	76	59.7	57.9	26.88	8.9	15.18	31.1
			placebo + insulin	70	56.1	70.0	25.99	8.9	12.34	28.1
Neal *et al*.^37^	18	Stable	canagliflozin 300 mg qd + insulin ± OADs	690	63.0^a^	65.0	33.3	8.3	16.3	60.0^a^
			placebo + insulin ± OADs	690	63.0^a^	66.0	33.1	8.3	16.0	58.0^a^
Rosenstock *et al*.^43^	18	Stable	empagliflozin 25 mg qd + insulin ± metformin	189	58.0	44.0	35.0	8.3	NR	92.9
			placebo + insulin ± metformin	188	55.3	40.0	34.7	8.3	NR	93.1
Rosenstock *et al*.^44^	18	Stable	empagliflozin 25 mg qd + insulin ± metformin ± SU	155	59.9	60.0	32.7	8.3	NR	48.4
			placebo + insulin ± metformin ± SU	170	58.1	53.0	31.8	8.2	NR	47.8
Wilding *et al*.^51^	12	Stable	dapagliflozin 10 mg qd + insulin ± metformin ± TZD	24	55.7	54.2	35.5	8.4	11.8	93.0
			placebo + insulin ± metformin ± TZD	23	58.4	69.6	34.8	8.4	13.8	80.0
Wilding *et al*.^52^	24	Stable	dapagliflozin 10 mg qd + insulin ± OADs	194	59.3	44.8	33.4	8.6	14.2	78.0
			placebo + insulin ± OADs	193	58.8	49.5	33.1	8.5	13.5	73.7
NCT02096705^58^	24	NR	dapagliflozin 10 mg qd + insulin	139	56.5	47.5	NR	NR	NR	NR
			placebo + insulin	133	58.6	48.1	NR	NR	NR	NR
Insulin plus thiazolidinedione										
Asnani *et al*.^14^	16	Titration	pioglitazone 30 mg qd + insulin ± OADs	8	59.0	NR	NR	10.0	17.0	NR
			placebo + insulin ± OADs	8	57.0	NR	NR	8.7	11.0	NR
Buse *et al*.^17^	26	Titration	troglitazone 400 mg qd + insulin	76	58.0	50.0	34.8	9.0	NR	NR
			placebo + insulin	71	57.0	49.0	34.5	9.0	NR	NR
Hanefeld *et al*.^24^	24	Titration	pioglitazone 15 mg bid + glargine + metformin	39	63.3	66.7	33.1	7.3	11.0	34.9
			placebo + glargine + metformin	42	64.2	54.8	31.8	7.4	12.3	36.6
Henriksen *et al*.^25^	26	Stable	pioglitazone 45 mg qd + insulin	102	60.1	69.0	33.2	8.7	13.8	78.4
			balaglitazone 20 mg qd + insulin	97	60.5	55.0	34.1	8.5	14.7	80.2
			placebo + insulin	106	60.9	62.0	33.9	8.5	12.6	75.2
Hodis *et al*.^27^	24	Titration	troglitazone 400 mg qd + insulin	142	52.4	33.1	32.1	9.9	9.8	51.5
			placebo + insulin	134	52.6	32.1	31.1	9.7	9.7	54.5
Hollander *et al*.^28^	24	Titration	rosiglitazone 2 mg bid + insulin	189	52.6	48.1	33.7	9.0	13.0	73.5
			placebo + insulin	186	53.8	46.2	33.0	9.1	12.6	80.3
Mattoo *et al*.^35^	24	Titration	pioglitazone 30 mg qd + insulin ± OADs	142	58.8	43.7	32.5	8.9	13.62	NR
			placebo + insulin ± OADs	147	58.9	42.9	31.8	8.8	13.41	NR
Naka *et al*.^36^	24	NR	rosiglitazone 4 mg qd + insulin	17	64.7	17.6	28.8	8.8	20.1	48.4
			insulin	14	67.3	28.6	29.0	8.8	17.1	47.1
Raskin *et al*.^39^	26	Stable	rosiglitazone 4 mg bid + insulin	103	57.7	54.4	32.3	9.0	12.5	77.7
			placebo + insulin	104	55.6	55.8	32.7	8.9	11.1	70.1
Reynolds *et al*.^40^	24	Stable	rosiglitazone 4 mg per day + insulin	8	NR	NR	36.4	8.0	NR	73.1
			placebo + insulin	10	NR	NR	36.3	9.8	NR	72
Shah *et al*.^49^	12–16	Titration	pioglitazone 45 mg per day + insulin	12	58.0	84.0	36.7	7.6	NR	105.0
			placebo + insulin	13	58.0	84.0	36.7	7.8	NR	114.0
Yasunari *et al*.^53^	48	Titration	pioglitazone 15 mg bid + insulin + OADs	22	56.0	81.8	25.5	8.6	13.4	37.2
			insulin ± OADs	26	57.2	69.2	26.9	8.6	14.8	39.6
Yilmaz *et al*.^54^	24	Titration	rosiglitazone 8 mg qd + insulin	15	57.6	53.3	30.7	9.6	12.1	41.9
			insulin	19	61.5	36.8	28.2	8.7	17.9	42.7

Abbreviations: NR, not reported; OAD, oral antidiabetic drugs; SU, sulfonylurea; TZD, thiazolidinedione; DPP4i, dipeptidyl peptidase-4 inhibitor; bid, twice a day; qd, once a day; qw, once weekly; ^a^, median; ‘insulin’ denotes multiple types of insulin preparations.

### Quality assessment of the included studies

Twenty-seven^15,18,20,22–24,26,28,30,31,33,34,36,38,40,41,43,44,47–49,51,54,57–59,61^ (54%) and twenty-three^13,14,20–24,26,28,32,34,36,40,49–51,53,54,57–61^ (46%) of the 50 studies did not describe their methods of generating a random number and of allocation concealment, respectively. In the assessment for the blinding of patients and personnel, 8 trials^20,21,33,36,46,53,54,56^ (16%) were evaluated as high-risk, as they were open-label trials. For the risk of incomplete outcome data, most studies^12–16,18,20,22,24–35,37,38,40–45,47,48,50–52,55–61^ (80%) were rated as low-risk. Three studies^57–59^ were considered open to a high risk of selective reporting bias since we used their data from the Clinicaltrials.gov website only, and those data were not regarded as complete reports. Most of the trials^12–22,24–48,50–56,60,61^ (90%) had balanced baseline characteristics between the treatment groups. The detailed results of the assessment are found in Supplementary Figure 1.

### Efficacy outcomes

We initially compared the primary outcome values from the common control group among the included studies. The pooled weighted mean changes in HbA1c from baseline in the control group were −0.16% (95% CI, −0.33% to 0.01%), −0.55% (−0.82% to −0.27%), −0.05% (−0.21% to 0.10%), and −0.11% (−0.32% to 0.09%) in the DPP4i, GLP-1RA, SGLT2i, and TZD studies, respectively, with a large extent of within-group heterogeneity (I^2^ = 94.5%, 97.9%, 94.7%, and 80.5%, respectively) (Supplementary Figure 2A). Considerable between-group heterogeneity was also observed (*P* < 0.0001). Greater reduction of HbA1c in the control group was noted in some studies with GLP-1RA or TZD treatments and those also adopted some type of insulin titration method. An inherited relationship was observed between the change in the HbA1c in the control group and the treatment difference in the changes of the HbA1c (*P* = 0.02) (Supplementary Figure 2B). Therefore, we took this modification effect into account by adjusting for the value in the control group. Age, sex, and BMI at baseline also had significant relationships with the treatment difference in the change of HbA1c (*P* < 0.05 for all) (Supplementary Figure 3).

In the adjusted analysis, all 4 add-on anti-diabetic agents showed a greater reduction of HbA1c compared to the control group: DPP4i, −0.54% (95% CrI, −0.68% to −0.39%); GLP-1RA, −0.84% (−1.00% to −0.69%); SGLT2i, −0.66% (−0.84% to −0.48%); and TZD, −0.73% (−0.93% to −0.52%) (Table 2). The difference in change of HbA1c between the GLP-1RA and DPP4i groups was significant (−0.30%; −0.52% to −0.09%). GLP-1RA showed the highest probability of being the best for glycemic control (Table 3). The unadjusted analysis also suggested a similar trend but failed to show a significant difference between GLP-1RA and DPP4i (Supplementary Table 1). The results were confirmed by sensitivity analyses (Supplementary Tables 2 and 3).

**Table 2:** Pairwise results of comparisons between antidiabetic agents as an add-on to pre-existing insulin therapy from network meta-analyses adjusted by study-level covariates.

Difference in mean change of HbA1c from baseline (95% credible interval), %				
Control	−0.54(−0.68, −0.39)	−0.84(−1.00,−0.69)	−0.66(−0.84,−0.48)	−0.73(−0.93,−0.52)
—	DPP4i	−0.30(−0.52,−0.09)	−0.11(−0.36, 0.12)	−0.18(−0.44, 0.07)
—	—	GLP-1RA	0.19(−0.06, 0.43)	0.11(−0.16, 0.40)
—	—	—	SGLT2i	−0.07(−0.33, 0.20)
—	—	—	—	TZD
**Difference in mean change of FPG from baseline (95% credible interval), mg/dL [mmol/L]**				
Control	−11.42(−15.47, −7.36) [−0.63(−0.86, −0.41)]	−9.96(−14.55, −5.38) [−0.55(−0.81, −0.30)]	−24.14(−29.64, −18.54) [−1.34(−1.65, −1.03)]	−19.57(−24.78, −14.25) [−1.09(−1.38, −0.79)]
—	DPP4i	1.48(−4.58, 7.42) [0.08(−0.25, 0.41)]	−12.72(−19.53, −5.82) [−0.71(−1.08, −0.32)]	−8.12(−14.79, −1.37) [−0.45(−0.82, −0.08)]
—	—	GLP-1RA	−14.17(−21.69, −6.73) [−0.79(−1.20, −0.37)]	−9.61(−16.65, 2.38) [−0.53(−0.92, 0.13)]
—	—	—	SGLT2i	4.61(−2.93, 12.11) [0.26(−0.16, 0.67)]
—	—	—	—	TZD
**Difference in mean change of body weight from baseline (95% credible interval), kg**				
Control	−0.10(−0.83, 0.64)	−2.20(−2.87, −1.57)	−1.75(−2.65, −0.85)	2.58(1.63, 3.48)
—	DPP4i	−2.11(−3.11, −1.15)	−1.65(−2.81, −0.47)	2.67(1.46, 3.82)
—	—	GLP-1RA	0.46(−0.64, 1.58)	4.78(3.63, 5.90)
—	—	—	SGLT2i	4.32(2.98, 5.60)
—	—	—	—	TZD
**Relative proportion of participants attaining HbA1c levels of <7% (95% credible interval)**				
Control	2.68(1.80, 3.81)	3.70(2.89, 4.67)	1.83(0.64, 3.90)	2.18(1.12, 3.58)
—	DPP4i	1.43(0.93, 2.15)	0.72(0.21, 1.69)	0.84(0.39, 1.51)
—	—	GLP-1RA	0.50(0.16, 1.11)	0.60(0.29, 1.02)
—	—	—	SGLT2i	1.48(0.42, 3.81)
—	—	—	—	TZD
**Difference in mean change of daily insulin dose from baseline (95% credible interval), IU/day**				
Control	−3.87(−7.70, −0.10)	−8.61(−12.34, −5.00)	−4.64(−9.62, 0.32)	−11.97(−15.48, −8.41)
—	DPP4i	−4.76(−9.68, 0.16)	−0.79(−6.99, 5.51)	−8.09(−13.26, −2.88)
—	—	GLP-1RA	3.94(−2.23, 10.14)	−3.36(−8.33, 1.74)
—	—	—	SGLT2i	−7.29(−13.45, −1.12)
—	—	—	—	TZD
**Relative risk of hypoglycemia (95% credible interval)**				
Control	1.06(0.85, 1.32)	1.37(1.08, 1.71)	1.22(0.88, 1.67)	1.94(1.39, 2.62)
—	DPP4i	1.30(0.94, 1.77)	1.15(0.78, 1.70)	1.83(1.23, 2.65)
—	—	GLP-1RA	0.89(0.60,1.32)	1.41(0.95, 2.06)
—	—	—	SGLT2i	1.59(1.01, 2.49)
—	—	—	—	TZD

Abbreviations: DPP4i, dipeptidyl peptidase-4 inhibitor; GLP-1RA, glucagon like peptide-1 receptor agonists; SGLT2i, sodium-glucose co-transporter 2 inhibitor; TZD, thiazolidinedione; FPG, fasting plasma glucose.

**Table 3:** Probabilities (%) of being the highest-ranked group for each study outcome

	DPP4i	GLP-1RA	SGLT2i	TZD
Reduction of HbA1c from baseline	0.13	77.14	4.21	18.52
Reduction of FPG from baseline	0.00	0.00	88.94	11.06
Reduction of body weight from baseline	0.00	79.64	20.36	0.00
Proportion of HbA1c <7%	4.64	88.76	4.14	2.46
Reduction of insulin dose from baseline	0.00	9.55	0.79	89.61
Risk of hypoglycemia	0.00	3.82	1.88	94.24

Abbreviations: DPP4i, dipeptidyl peptidase-4 inhibitor; GLP-1RA, glucagon like peptide-1 receptor agonists; SGLT2i, sodium-glucose co-transporter 2 inhibitor; TZD, thiazolidinedione; FPG, fasting plasma glucose.

Forty-one studies^12,13,15–18,22,24–27,29–36,38,39,41–48,50–61^ assessed changes in the FPG level from baseline. All groups showed a significantly greater reduction in FPG than the control group in the adjusted analysis: DPP4i, −11.42 mg/dL (−15.47 to −7.36 mg/dL) [−0.63 mmol/L (−0.86 to −0.41 mmol/L)]; GLP1-RA, −9.96 mg/dL (−14.55 to −5.38 mg/dL) [−0.55 mmol/L (−0.81 to −0.30 mmol/L)]; SGLT2i, −24.14 mg/dL (−29.64 to −18.54 mg/dL) [−1.34 mmol/L (−1.65 to −1.03 mmol/L)]; TZD, −19.57 mg/dL (−24.78 to −14.25 mg/dL) [−1.09 mmol/L (−1.38 to −0.79 mmol/L)] (Table 2). SGLT2i showed a significantly greater reduction in FPG compared to DPP4i and GLP1-RA. SGLT2i was ranked as the best treatment for FPG reduction (Table 3). Sensitivity analysis with missing covariate imputation was not required for this outcome.

Thirty-eight trials^12,13,15–18,20–22,25,27,30,32–36,40–56,58–61^ assessed changes in body weight from baseline. GLP-1RA and SGLT2i decreased body weight significantly more than DPP4i (−2.11 kg, −3.11 to −1.15 kg; and −1.65 kg, −2.81 to −0.47 kg, respectively), whereas TZD showed an increase in body weight compared to DPP4i (2.67 kg, 1.46 to 3.82 kg) (Table 2). The difference between the DPP4i and the control group was not significant. GLP-1RA had the highest probability of being ranked the first for body weight reduction, followed by SGLT2i (Table 3). The results were consistent in the sensitivity analysis after the imputation of missing covariates (Supplementary Table 2).

Thirty-two trials^12,15,16,18,20,21,23,24,26,28,30–35,37,38,41–48,50,51,55,56,59,61^ described the proportion of participants attaining the target HbA1c level ($<$7.0%). GLP-1RA, DPP4i, and TZD groups showed a significantly higher proportion of achieving the target HbA1c level than the control (RR = 3.70, 2.89–4.67; RR = 2.68, 1.80–3.81; and RR = 2.18, 1.12–3.58, respectively), among which GLP-1RA showed the highest probability of being the best for reaching the target HbA1c level (Tables 2 and 3). Only 2 SGLT2i studies^37,51^ were included in the covariate-adjusted analysis, because the other SGLT2i studies had some missing covariates. When we reanalyzed the results with an imputation of the missing covariates, all 4 groups showed a significant increase in the rate of achieving the target HbA1c level compared to the control group (Supplementary Table 2).

For the change in insulin doses from baseline, 34 studies^13,15,16,18,20–22,24,25,27,28,32–34,39–42,44–56,58,59,61^ were included in the analysis. DPP4i, GLP-1RA, and TZD showed a significant reduction in the insulin dose compared to the control group (−3.87 IU/day, −7.70 to −0.10 IU/day; −8.61 IU/day, −12.34 to −5.00 IU/day; and −11.97 IU/day, −15.48 to −8.41 IU/day, respectively) (Table 2). The insulin-sparing effects of TZD were significantly greater than those of DPP4i and SGLT2i. TZD was ranked the highest in terms of its insulin-sparing effect, followed by GLP-1RA (Table 3). A sensitivity analysis after imputing missing covariates confirmed the results (Supplementary Table 2).

### Hypoglycemia

Thirty-four studies^12,13,15,16,18,20–22,24–26,29–31,33–35,38,39,41–45,48,50–52,55–57,59–61^ were included in the analysis. The risk of hypoglycemia was not greater in the DPP4i or SGLT2i groups than in the control group. Hypoglycemia risk with TZD was found to be significantly greater than with DPP4i and SGLT2i (RR = 1.83, 1.23–2.65 and RR = 1.59, 1.01–2.49, respectively) (Table 2). The TZD group was ranked the highest for risk of hypoglycemia (Table 3). The risk of hypoglycemia was not associated with any potential covariates, and therefore no adjustment was required for this analysis.

## Discussion

Through a current systematic review and network meta-analysis based on 50 RCTs including 15,494 participants, we report the best available evidence comparing the efficacy and safety among different types of non-insulin anti-diabetic agents as an add-on to pre-existing insulin therapy in patients with inadequately controlled T2DM. The principal findings of our study are as follows: (1) GLP-1RA showed the greatest effect on HbA1c reduction, followed by TZD, SGLT2i, and DPP4i; (2) the reduction in FPG was higher with SGLT2i than with DPP4i and GLP1-RA; (3) GLP-1RA and SGLT2i were associated with body weight reduction, whereas TZD increased body weight; (4) TZD and GLP-1RA reduced total daily insulin requirements; (5) the risk of hypoglycemia was increased with TZD and GLP-1RA.

The studies included in our analysis had different baseline characteristics and showed significant within-group and between-group heterogeneity. We found that age, sex, and BMI at baseline were associated with placebo-subtracted HbA1c reduction in a linear relationship; therefore, we adjusted for those potential confounding factors as covariates. In addition, since it was not possible to adjust for various methods of insulin titration among the included studies, we instead adjusted for the treatment effects of the control group. After adjustment for the covariates, GLP-1RA was at the top rank of the hierarchy. In addition, the results were similar in the sensitivity analyses, with GLP-1RA showing the highest probability of being the best for glycemic control. Consistent with the primary outcome, GLP-1RA also showed the highest rank for achieving HbA1c levels <7.0%. This might be explained by the complementary effects of GLP-1RA with insulin therapy^62^.

The FPG-lowering efficacy of SGLT2i was significantly greater than that of DPP4i and GLP-1RA, and tended to be greater than that of TZD. SGLT2i drugs act on the kidneys and increase glucose excretion through the urine, which is mediated by an insulin-independent mechanism; therefore, they reduce both FPG and postprandial glucose (PPG)^63^. In contrast, GLP-1RA agents have different FPG- and PPG-lowering efficacy, depending on their duration of action. Short-acting GLP-1RA drugs drastically decelerate gastric emptying, leading to a marked reduction in the PPG excursion. In contrast, long-acting GLP-1RA drugs have little effect on gastric emptying, but their long-lasting effect on pancreatic beta-cell and alpha-cell function contributes to the predominant reduction in FPG levels^64^. Because about half of the GLP-1RA studies used long-acting GLP-1RA agents, and the rest of the studies used short-acting GLP-1RA agents, the FPG-lowering effect of add-on GLP-1RA might be mixed.

Weight gain is commonly associated with insulin therapy, particularly with intensive insulin therapy^65^. In this network meta-analysis, GLP-1RA appeared to be the most effective for weight reduction, which is attributed to decreasing appetite and promoting satiety^64^. SGLT2i was ranked lower than GLP-1RA, possibly because the weight-loss effect of SGLT2i is compensated by increased appetite and calorie intake^66,67^. Overall, the reported effects of weight loss with GLP-1RA and SGLT2i, weight gain with TZD, and no effect for DPP4i were preserved when combined with preexisting insulin therapy in patients with T2DM^62,63,68^.

The insulin-sparing effect, which was pre-defined as the reduction in the total required daily dose of insulin from baseline, was greater in TZD and GLP-1RA. The greater potency of GLP-1RA and TZD in glycemic control and their insulin-sensitizing effects may contribute to reducing insulin dosage^62,69^. Differences in the insulin titration algorithm may also have contributed to the different insulin-sparing effect across the studies. More than half of the studies of TZD and GLP-1RA adopted insulin dose titration regimens, whereas most studies of SGLT2i and DPP4i adopted stable insulin dose regimens. The placebo-subtracted insulin-sparing effects in studies with active insulin dose titration tend to be greater than in studies with stable insulin dose regimens.

In the current study, SGLT2i and DPP4i were suggested to have a potentially lower risk of hypoglycemia than the other 2 agents. In general, SGLT2i drugs do not induce hypoglycemia because they increase plasma glucagon concentrations and decrease plasma insulin concentrations^70,71^. DPP4i drugs also have a minimal risk of hypoglycemia due to their glucose-dependent mechanism of action regarding the regulation of insulin and glucagon secretion^62^. Theoretically, TZD and GLP-1RA drugs seldom provoke hypoglycemia by themselves unless combined with insulinotrophic agents^64,72^. In this study, however, TZD and GLP-1RA agents showed an increased risk of hypoglycemia in comparison to the control group. The greater glucose-lowering potency of TZD and GLP-1RA and adopting an active insulin dose titration regimen might contribute to the increased risk. Because the definitions of hypoglycemia varied widely across the included studies, the comparative risk of hypoglycemia among these agents must be carefully interpreted.

The study has several limitations. First, the current study is based on indirect comparisons using a common control. Network meta-analyses are typically required to address inconsistencies between the results of direct and indirect comparisons. However, since no study has conducted head-to-head comparisons among GLP-1RA, DPP4i, SGLT2i, and TZD, it was not possible to evaluate consistency between the results of direct and indirect comparisons. We therefore primarily explored the heterogeneity among the values of HbA1c reduction in the control group, which possibly indicates a lack of comparability among the included studies. We then identified as many potential confounders as possible and adjusted for their effect in the final analysis. Second, the patients in the studies included in this study used various baseline insulin regimens, but we could not isolate the effects of the studied medications on top of the basal insulin therapy regimen, which is the most common practice of insulin therapy at present. Finally, due to lack of information, we could not assess drug-specific safety outcomes, such as gastrointestinal adverse events and urinary tract infections.

Based on the evidence from our network meta-analysis, we may suggest patient-centered guidance for non-insulin anti-diabetic agents in patients with inadequately controlled T2DM on pre-existing insulin therapy. For those who need to reduce both HbA1c and body weight, GLP-1RA might be an optimal choice. Fixed-ratio combinations of insulin and GLP-1RA are currently available to improve glycemic control and to minimize the risk of weight gain^33,56^. For patients who need to lower their FPG level, SGLT2i might be a good treatment option due to its superior FPG-lowering efficacy without increasing weight gain or hypoglycemia. It also has the advantage of body weight reduction. Even though DPP4i was relatively less efficacious for HbA1c reduction, it rarely increased the risk of hypoglycemia. Therefore, DPP4i may be considered for patients with relatively less severe hyperglycemia but with a high risk of hypoglycemia. Lastly, TZD might be taken into consideration in patients with severe insulin resistance requiring high doses of insulin.

Since a previous study which compared insulin monotherapy with the addition of various oral glucose-lowering agents to insulin did not present the comparative efficacy among non-insulin anti-diabetic agents in patients with inadequately controlled T2DM with insulin therapy^73^, our systematic review and network meta-analysis provides evidence-based suggestions for selecting an appropriate non-insulin antidiabetic agent based on patients’ clinical characteristics. Nonetheless, RCTs directly comparing the efficacy and safety of these agents should be undertaken.

## Electronic supplementary material

Supplementary information

PRISMA NMA checklist
