# Supplementary material for: Comparison of non-insulin antidiabetic agents as an add-on drug to insulin therapy in type 2 diabetes: a network meta-analysis
Source: Sci Rep. 2018 Mar 6;8:4095. doi: 10.1038/s41598-018-22443-1 (PMC5840350; doi:10.1038/s41598-018-22443-1)
Supplement: Supplementary file 1 — Supplementary information [file 41598_2018_22443_MOESM1_ESM.pdf]

Comparison of non-insulin antidiabetic agents as an add-on drug to insulin therapy in type 2 diabetes: a network meta-analysis

Jeong-Hwa Yoon[1]*, Se Hee Min [2]*, Chang Ho Ahn[2], Young Min Cho[2]**, Seokyung Hahn[3]**

[1]Interdisciplinary Program in Medical Informatics, Seoul National University College of Medicine, Seoul, South Korea

[2]Division of Endocrinology and Metabolism, Department of Internal Medicine, Seoul National University College of Medicine, Seoul, South Korea

[3]Department of Medicine, Seoul National University College of Medicine, Seoul, South Korea

* J.-H.Y. and S.H.M. contributed equally to this work

** S.H. and Y.M.C. are co-corresponding authors

Correspondence:

Seokyung Hahn, PhD, Co-corresponding author

Department of Medicine, Seoul National University College of Medicine

103 Daehak-ro, Jongno-gu, Seoul 03080, Korea

Tel: +82-2-740-8911

Fax: +82-2-743-8361

E-mail: hahns@snu.ac.kr

Young Min Cho, MD, PhD Co-corresponding author

Department of Internal Medicine, Seoul National University College of Medicine

101 Daehak-ro, Jongno-gu, Seoul 03080, Korea

Tel: +82-2-2072-1965

Fax: +82-2-762-9662

E-mail: ymchomd@snu.ac.kr

Supplementary Appendix 1

Title: Comparison among a variety of insulin combination therapies: network meta-analysis protocol

1. **Objectives**: The primary objective of this review is to evaluate the glycemic control effects of DPP4 inhibitors, GLP-1 analogues, SGLT2 inhibitors, TZD, and placebo when added onto pre-existing insulin therapy.

2. The reporting of the review will follow the preferred reporting items for the PRISMA extension statement for the reporting of systematic reviews incorporating network meta-analyses.

3. **Protocol and registration**: Methods of the analysis and inclusion criteria were specified in advance and documented in this protocol.

4. **Eligibility criteria**

   1) PICOS (population or patient group, intervention, comparison intervention, outcome, study design)

   - Patient: Type 2 diabetes patients
   - Intervention and comparator:

     Insulin ＋ none, SGLT2i, DPP4i, TZD, GLP-1 analogue, or placebo

     ± oral antidiabetic medications

     (Any comparison that evaluates the efficacy of these drugs is allowed.)
   - Outcomes:

- Primary: Δ HbA1c from baseline to follow-up date
- Secondary: Δ FPG from baseline to follow-up date, Δ body weight from baseline to follow-up date and the risk of hypoglycemia

- Study design: randomized controlled trials, at least 12 weeks' study duration

2) Inclusion and exclusion criteria

(1) Adult patients (aged ≥18 years) of both genders with type 2 diabetes, whose HbA1c levels are above 6.5%.

(2) Studies of the comparative efficacy of SGLT2i, DPP4i, TZD, GLP-1 analogue, and placebo (or none) combined with insulin

(3) Prospective RCTs with follow-up periods >12 weeks

(4) Each trial reporting outcomes with mean and standard deviation (or 95% CI) of Δ HbA1c (endpoint HbA1c minus baseline HbA1c) in each group

(5) Baseline HbA1C level should be elucidated in each trial.

(6) Published in English

(7) No limitation for publication date

(8) Exclude extended study periods

(9) Exclude studies of patients treated with an insulin pump.

(10) Exclude post-hoc analyses

5. **Information sources:** Searching electronic databases. This search was applied to Medline, Embase, the Cochrane Controlled Trials Register, and ClinicalTrials.gov.

6. **Study selection**: Study selection will be performed independently by 2 investigators, and any disagreements will be resolved by consensus.

7. **Data extraction (clinical record form)**

   1) Study information:

      Study name, the first author name, published year, study design, study duration (weeks)

   2) Treatment information for arms of each study:

      Type of DPP4 inhibitor + insulin-based therapy

      Type of SGLT2 inhibitor + insulin-based therapy

      Type of TZD + insulin-based therapy

      Type of GLP-1 analogue + insulin-based therapy

      Placebo + insulin-based therapy

      Ex)) Sitagliptin (100 or 50 mg) + insulin vs. Placebo + insulin

   3) Patient information:

      Number of randomized participants

      Mean duration of diabetes (SD), years

      Mean age (SD), years

      Proportion of men, %

      Mean baseline BMI (SD), kg/m^2

      Mean baseline HbA1c level (SD), %

   4) Outcomes:

      - Efficacy

Mean change in HbA1c level (SE), %

Mean change in fasting plasma glucose levels (SD), mg/dL

Mean change in body weight (SD), kg

Between-group difference (mean and SE) for each value

- Safety

Proportion of risk of hypoglycemia

8. Other principles for data extraction

1) For dose-ranging, data with only an approved dose of a DPP4 inhibitor (or SGLT2 inhibitor, TZD, or GLP-1 analogue) will be selected for the analysis.

2) If there are 2 or more approved doses, data for the maximum approved dose will be selected.

3) If the trial is not a dose-ranging study, data with the dosage used in each trial will be extracted.

4) For extended studies, the results of the primary treatment duration will be used for analysis.

5) If the study used the least square mean difference adjusted by baseline covariates, the data will be extracted. However, if the outcomes were not presented in terms of covariate-adjusted values, the intergroup difference will be derived by calculating the arithmetical mean difference from the treatment and placebo group.

6) Any disagreements between reviewers will be resolved by consensus.

9. Quality assessment

1) Concealment of allocation

2) Blind methods

3) Selective reporting

4) Whether the analysis followed the intention-to-treat principle

5) Other sources of bias (comparability of participants' baseline characteristics)

Supplementary Appendix 2

Example of search term strategy (MEDLINE)

#1 glucagon like peptide 1/agonists[Title/Abstract] or glucagon-like peptide 1 receptor agonist[Title/Abstract] or Exenatide[Title/Abstract] or AC2993[Title/Abstract] or Lixisenatide[Title/Abstract] or ZP10A peptide[Title/Abstract] or ave0010[Title/Abstract] or Liraglutide[Title/Abstract] or nn2211[Title/Abstract] or Albiglutide[Title/Abstract] or LY 2189265[Title/Abstract] or Dulaglutide[Title/Abstract] or LY 2189265[Title/Abstract] or Taspoglutide[Title/Abstract] or R1583[Title/Abstract] or BIM 51077[Title/Abstract] or ZP10A peptide[Title/Abstract]

#2 dipeptidyl peptidase iv inhibitors[Title/Abstract] OR dipeptidyl peptidase iv inhibitors[Title/Abstract] OR dipeptidyl peptidase-4 inhibitor[Title/Abstract] OR dipeptidyl peptidase 4 inhibitor[Title/Abstract] OR DPP4[Title/Abstract] OR DPP-4[Title/Abstract] OR Sitagliptin[Title/Abstract] OR MK-0431[Title/Abstract] OR Vildagliptin[Title/Abstract] OR LAF237[Title/Abstract] OR Dutogliptin[Title/Abstract] OR PHX1149[Title/Abstract] OR Saxagliptin[Title/Abstract] OR BMS-477118[Title/Abstract] OR Linagliptin[Title/Abstract] OR BI1356[Title/Abstract] OR Alogliptin[Title/Abstract] OR SYR-322[Title/Abstract] OR Gemigliptin[Title/Abstract] OR LC15-0444[Title/Abstract] OR Teneg liptin[Title/Abstract] OR MP-513[Title/Abstract] OR Anagliptin[Title/Abstract] OR SK-0403[Title/Abstract] OR Gosogliptin[Title/Abstract] OR PF-734200[Title/Abstract] OR Evogliptin[Title/Abstract] OR DA-1229[Title/Abstract]

#3 sodium glucose transporter 2[Title/Abstract] OR sodium-glucose transporter 2[Title/Abstract] OR SGLT2[Title/Abstract] OR SGLT-2[Title/Abstract] OR Dapagliflozin[Title/Abstract] OR BMS-512148[Title/Abstract] OR Canagliflozin[Title/Abstract] OR TA-7284[Title/Abstract] OR Empagliflozin[Title/Abstract] OR BI-10773[Title/Abstract] OR Ipragliflozin[Title/Abstract] OR ASP1941[Title/Abstract] OR Luseogliflozin[Title/Abstract] OR TS-071[Title/Abstract] OR Tofogliflozin[Title/Abstract] OR CSG452[Title/Abstract] OR R7201[Title/Abstract] OR RG7201[Title/Abstract] OR Ertugliflozin[Title/Abstract] OR MK-8835[Title/Abstract] OR PF-04971729[Title/Abstract]

#4 pioglitazone[Title/Abstract] OR lobeglitazone[Title/Abstract] OR thiazolidinedione[Title/Abstract] OR thiazolidinediones[Title/Abstract] OR Troglitazone[Title/Abstract] OR rosiglitazone[Title/Abstract]

#5 insulin[Title/Abstract] or NPH[Title/Abstract] or glargine[Title/Abstract] or detemir[Title/Abstract] or degludec[Title/Abstract]

#6 (#1 or #2 or #3 or #4) and #5

#7 randomized controlled trial [pt]

#8 controlled clinical trial [pt]

#9 randomized [tiab]

#10 placebo [tiab]

#11 clinical trials as topic [mesh: noexp]

#12 randomly [tiab]

#13 trial [ti]

#14 #7 OR #8 OR #9 OR #10 OR #11 OR #12 OR #13

#15 animals [mh] NOT humans [mh]

#16 #14 not #15

#17 #6 and #16

Supplementary Figure 1. Risk of bias assessment

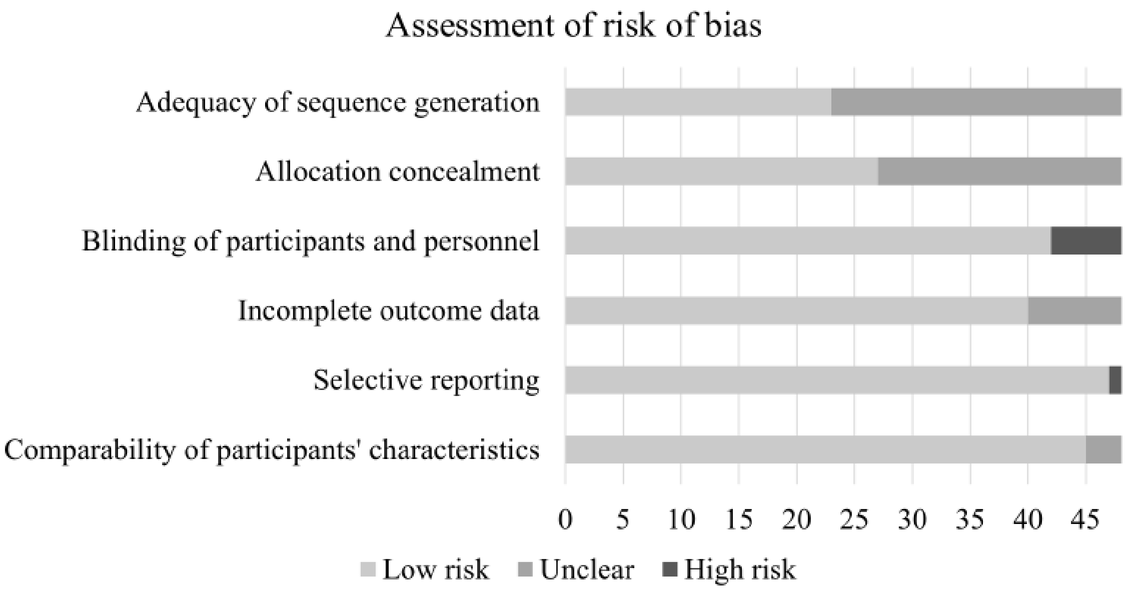

Supplementary Figure 2. A. Weighted mean change in HbA1c from baseline in control group. B. Meta-regression between the change in HbA1c in the control group and the treatment difference in the change of HbA1c.

A.

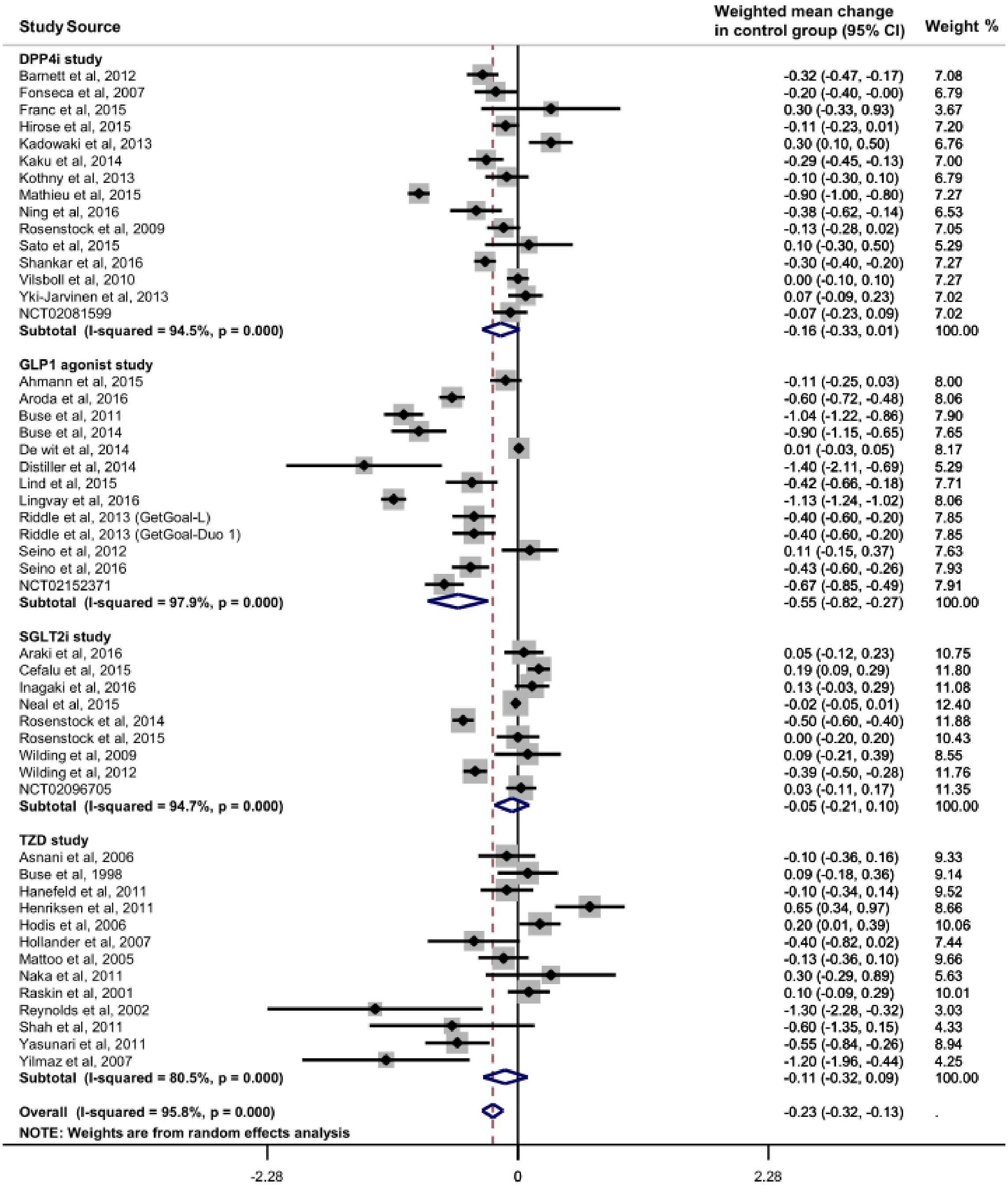

The change in HbA1c (%) from baseline in the controls groups of the DPP4i, GLP-1RA, SGLT2i, and TZD studies analyzed using a random-effects model. The squares indicate an individual study's effects and the size of the squares corresponds to the study's weight in the meta-analysis, with the horizontal lines extending from the symbols representing 95% CIs. The diamonds indicate the pooled estimates.(For the 3-arm trial, the treatment effect from only 1 treatment arm was presented.)

GLP-1RA, glucagon-like peptide-1 receptor agonists plus insulin; DPP4i, dipeptidyl peptidase-4 inhibitor plus insulin; SGLT2i, sodium-glucose co-transporter 2 inhibitor plus insulin; TZD thiazolidinedione plus insulin.

B.

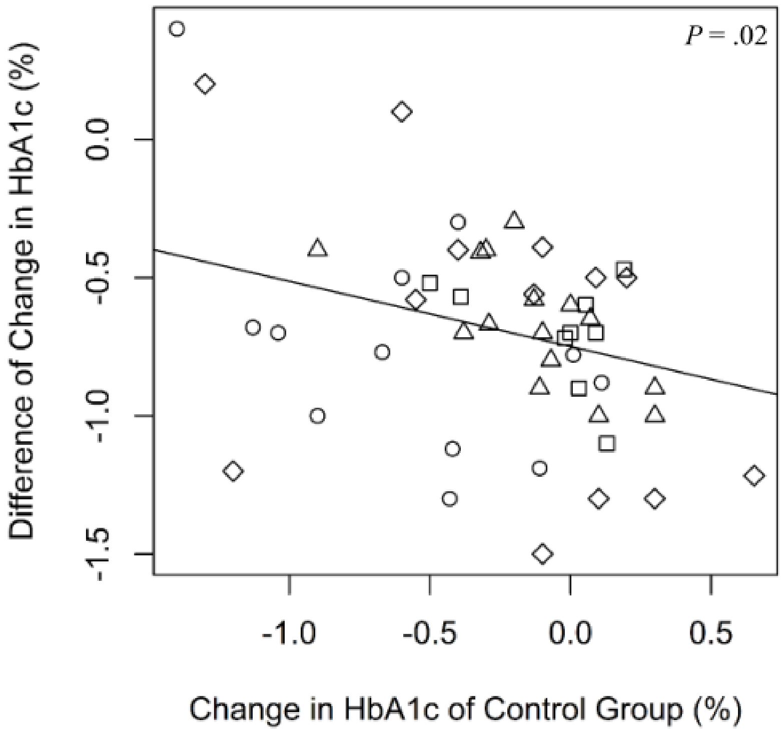

The triangles, circles, squares, and diamonds correspond to dipeptidyl peptidase-4 inhibitor studies, glucagon-like peptide-1 receptor agonists studies, sodium-glucose co-transporter 2 inhibitor studies and thiazolidinedione studies, respectively. Solid lines indicate the relationship between the change in the

HbA1c in the control group and the treatment difference in the changes of the HbA1c by meta-regression. The P value was obtained from meta-regression. (For the 3-arm trial, the treatment effect from only 1 treatment arm was presented.)

Supplementary Figure 3. Meta-regression between potential confounders and the treatment difference in the change of HbA1c.

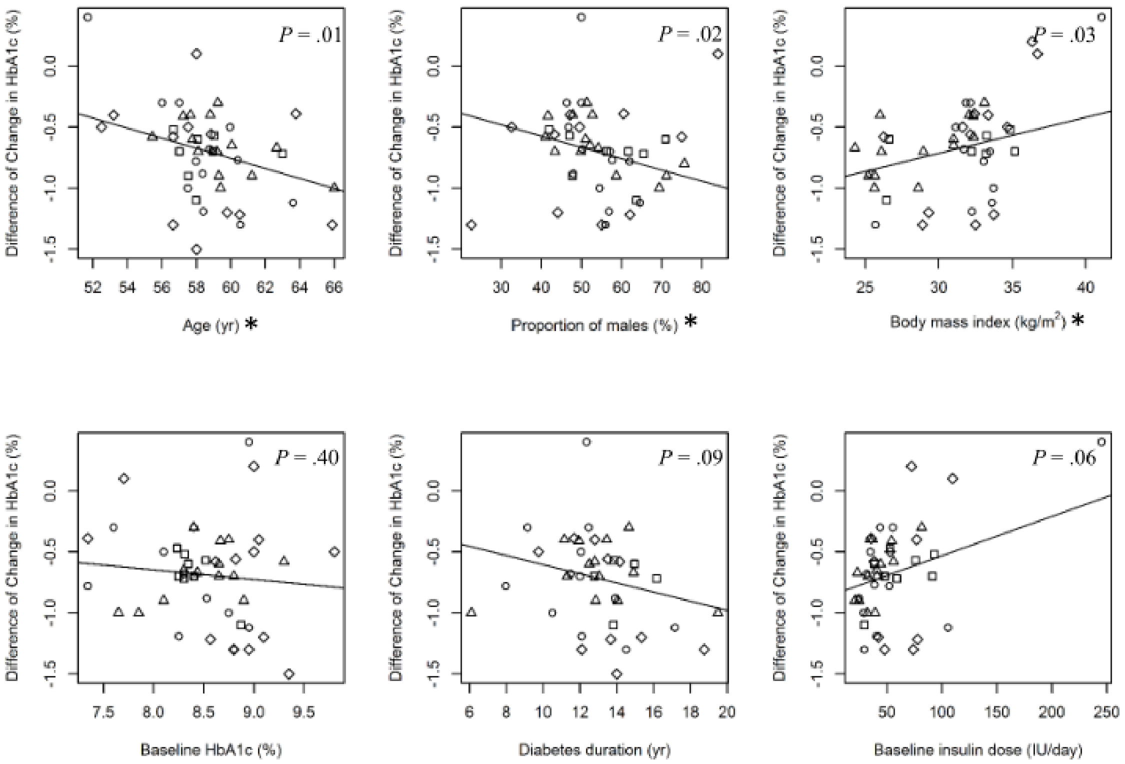

The triangles, circles, squares, and diamonds correspond to dipeptidyl peptidase-4 inhibitor studies, glucagon-like peptide-1 receptor agonist studies, sodium-glucose co-transporter 2 inhibitor studies and thiazolidinedione studies, respectively. Solid lines indicate the relationship between each potential covariate and the treatment difference in the changes of the HbA1c by meta-regression (for the 3-arm trial, 1 treatment effect is presented). P values were obtained from meta-regression. The star indicates the variable considered as covariates in the final model.

Supplementary Table 1. Pairwise results of comparisons between antidiabetic agents as an add-on to pre-existing insulin therapy from unadjusted network meta-analyses

**Difference in mean change of HbA1c from baseline (95% credible interval), %**				
Control	-0.63(-0.78, -0.49)	-0.76(-0.92, -0.60)	-0.69(-0.87, -0.52)	-0.74(-0.94, -0.54)
-	DPP4i	-0.13(-0.34, 0.09)	-0.06(-0.29, 0.17)	-0.11(-0.35, 0.14)
-	-	GLP-1RA	0.07(-0.17, 0.31)	0.03(-0.23, 0.28)
-	-	-	SGLT2i	-0.04(-0.31, 0.22)
-	-	-	-	TZD
**Difference in mean change of FPG from baseline (95% credible interval), mg/dL [mmol/L]**				
Control	-11.12(-15.25, -7.13)<br>[-0.62(-0.85, -0.40)]	-7.19(-11.42, -3.01)<br>[-0.40(-0.63, -0.17)]	-25.43(-30.89, -19.94)<br>[-1.41(-1.71, -1.11)]	-20.69(-26.03, -15.40)<br>[-1.15(-1.44, -0.85)]
-	DPP4i	3.93(-1.91, 9.79)<br>[0.22(-0.11, 0.54)]	-14.31(-21.08, -7.44)<br>[-0.79(-1.17, -0.41)]	-9.55(-16.24, -2.81)<br>[-0.53(-0.9, -0.16)]
-	-	GLP-1RA	-18.24(-25.03, -11.23)<br>[-1.01(-1.39, -0.62)]	-13.48(-20.26, -6.68)<br>[-0.75(-1.12, -0.37)]
-	-	-	SGLT2i	4.73(-2.82, 12.3)<br>[0.26(-0.16, 0.68)]
-	-	-	-	TZD
**Difference in mean change of body weight from baseline (95% credible interval), kg**				
Control	0.09(-0.77, 0.97)	-2.25(-3.06, -1.47)	-1.82(-2.84, -0.80)	1.89(0.90, 2.91)
-	DPP4i	-2.34(-3.55, -1.18)	-1.90(-3.26, -0.56)	1.80(0.48, 3.13)
-	-	GLP-1RA	0.43(-0.84, 1.73)	4.14(2.88, 5.43)
-	-	-	SGLT2i	3.71(2.25, 5.14)
-	-	-	-	TZD
**Relative proportion of participants attaining the HbA1c levels of <7% (95% credible interval)**				
Control	3.37(2.35, 4.71)	3.76(2.74, 5.04)	2.94(1.43, 5.03)	1.94(0.80, 3.72)
-	DPP4i	1.15(0.71, 1.74)	0.90(0.40, 1.66)	0.59(0.23, 1.20)
-	-	GLP-1RA	0.80(0.36, 1.42)	0.53(0.21, 1.05)
-	-	-	SGLT2i	0.73(0.24, 1.72)
-	-	-	-	TZD
**Difference in mean change of daily insulin dose from baseline (95% credible interval), IU/day**				
Control	-2.32(-5.97, 1.37)	-8.27(-11.74, -4.84)	-4.92(-9.59, -0.20)	-11.93(-15.21, -8.60)
-	DPP4i	-5.91(-11.00, -0.93)	-2.58(-8.57, 3.32)	-9.58(-14.53, -4.64)
-	-	GLP-1RA	3.35(-2.42, 9.13)	-3.64(-8.47, 1.14)
-	-	-	SGLT2i	-6.98(-12.73, -1.23)
-	-	-	-	TZD

Abbreviations: DPP4i, dipeptidyl peptidase-4 inhibitor; GLP-1RA, glucagon like peptide-1 receptor agonists; SGLT2i, sodium-glucose co-transporter 2 inhibitor; TZD, thiazolidinedione; FPG, fasting plasma glucose.

Supplementary Table 2. Pairwise results of comparisons between antidiabetic agents as an add-on to pre-existing insulin therapy from network meta-analyses adjusted by study-level covariates: sensitivity analysis with the imputation of missing covariates

**Difference in mean change of HbA1c from baseline (95% credible interval), %**				
Control	-0.56(-0.69, -0.42)	-0.84(-1.00, -0.70)	-0.64(-0.80,-0.49)	-0.72(-0.92, -0.53)
-	DPP4i	-0.29(-0.50, -0.08)	-0.08(-0.30, 0.12)	-0.17(-0.41, 0.08)
-	-	GLP-1RA	0.20(-0.02, 0.43)	0.12(-0.14, 0.39)
-	-	-	SGLT2i	-0.08(-0.32, 0.15)
-	-	-	-	TZD
**Difference in mean change of body weight from baseline (95% credible interval), kg**				
Control	-0.09(-0.96, 0.81)	-2.24(-3.03, -1.48)	-1.83(-2.84, -0.82)	1.91(0.91, 2.91)
-	DPP4i	-2.15(-3.35, -0.97)	-1.75(-3.10, -0.41)	2.00(0.65, 3.34)
-	-	GLP-1RA	0.40(-0.84, 1.70)	4.15(2.90, 5.43)
-	-	-	SGLT2i	3.75(2.32, 5.16)
-	-	-	-	TZD
**Relative proportion of participants attaining the HbA1c levels of <7% (95% credible interval)**				
Control	3.04(2.21, 4.12)	4.12(3.23, 5.25)	3.11(1.72, 4.97)	2.51(1.27, 4.26)
-	DPP4i	1.39(0.93, 2.01)	1.05(0.51, 1.85)	0.85(0.39, 1.54)
-	-	GLP-1RA	0.76(0.40, 1.26)	0.62(0.30, 1.07)
-	-	-	SGLT2i	0.86(0.37, 1.72)
-	-	-	-	TZD
**Difference in mean change of daily insulin dose from baseline (95% credible interval), IU/day**				
Control	-3.76(-7.72, 0.08)	-8.74(-12.41, -5.08)	-4.98(-9.61, -0.34)	-12.18(-15.48, -8.93)
-	DPP4i	-4.99(-9.88, 0.06)	-1.22(-7.11, 4.77)	-8.43(-13.49, -3.32)
-	-	GLP-1RA	3.76(-2.01, 9.62)	-3.45(-8.32, 1.53)
-	-	-	SGLT2i	-7.2(-12.90, -1.52)
-	-	-	-	TZD

Abbreviations: DPP4i, dipeptidyl peptidase-4 inhibitor; GLP-1RA, glucagon like peptide-1 receptor agonists; SGLT2i, sodium-glucose co-transporter 2 inhibitor; TZD, thiazolidinedione; FPG, fasting plasma glucose.

Supplementary Table 3. Pairwise results of comparisons between antidiabetic agents as an add-on to pre-existing insulin therapy from network meta-analyses adjusted by study-level covariates: sensitivity analysis using only placebo-controlled studies

**Difference in mean change of HbA1c from baseline (95% credible interval), %**				
Control	-0.58(-0.74, -0.42)	-0.92(-1.13, -0.72)	-0.65(-0.84, -0.46)	-0.69(-0.92, -0.45)
-	DPP4i	-0.34(-0.59, -0.09)	-0.07(-0.34, 0.20)	-0.11(-0.41, 0.21)
-	-	GLP-1RA	0.27(-0.01, 0.55)	0.23(-0.11, 0.58)
-	-	-	SGLT2i	-0.04(-0.33, 0.26)
-	-	-	-	TZD

Abbreviations: DPP4i, dipeptidyl peptidase-4 inhibitor; GLP-1RA, glucagon like peptide-1 receptor agonists; SGLT2i, sodium-glucose co-transporter 2 inhibitor; TZD, thiazolidinedione.
